# Dose–response relationship and predictive value of soluble B7‐DC in bronchoalveolar lavage fluid and risk of refractory Mycoplasma pneumoniae pneumonia in children

**DOI:** 10.1002/kjm2.12944

**Published:** 2025-02-13

**Authors:** Xue‐Hua Li, Jun‐Mei Xu

**Affiliations:** ^1^ Department of Pediatrics, Beijing Friendship Hospital Capital Medical University Beijing City China

**Keywords:** bronchoalveolar lavage fluid, column graph, predictive value, refractory mycoplasma pneumonia, soluble B7‐DC

## Abstract

This study was to investigate the clinical significance of soluble B7‐dendritic cell (sB7‐DC) concentration in bronchoalveolar lavage fluid (BALF) of children with Refractory Mycoplasma pneumoniae pneumonia (RMPP). A total of 298 patients with Mycoplasma pneumoniae pneumonia (MPP) were enrolled. Patients were divided into general MPP (GMPP) (*n* = 213) and RMPP groups (*n* = 85). Detection of sB7‐DC and serum inflammatory factors in BALF was performed by ELISA. The relationship between sB7‐DC and the risk of RMPP was assessed using restricted cubic spline (RCS) model. A base model for predicting RMPP was constructed using logistic regression analysis, and a compound model was created with the addition of sB7‐DC in the base model. ROC curves were plotted to evaluate the predictive value of the model. Column line plots were plotted to assess the contribution of each variable to the outcome event. Calibration curves were plotted and the Hosmer–Lemeshow test (HL test) was performed to assess the calibration performance of the model. Decision curve analysis (DCA) plots were plotted to assess determine whether sB7‐DC has clinical value. There was no statistical difference between sB7‐H3 and sB7‐H4 in the two groups (both *p* > 0.05). sB7‐DC levels were higher in the RMPP group than in the GMPP group (91.66 [77.36, 122.5] pg/ml vs. 64.87 [47.07, 86.46] pg/ml, *p* < 0.001). RCS analysis showed that the risk of RMPP gradually increased with the increase of sB7‐DC when sB7‐DC > 76.505 pg/ml. Both the base model and the compound model constructed with independent correlates of RMPP had some predictive value, and the models were well‐fitted. The column line graphs showed that the models had discriminative ability. Notably, the compound model had a higher predictive value, with a higher AUC value than the base model: 0.76 (0.65–0.87) versus 0.68 (0.54–0.81). The highest net benefit was close to 0.15 (only 0.1 in the base model). When the net benefit was >0, the high‐risk threshold took on a wide range of values. sB7‐DC in children with RMPP is an independent predictor of RMPP. sB7‐DC helps to improve quantitative prediction of RMPP risk and accurately guide medical decisions.

## 1 INTRODUCTION

Mycoplasma pneumoniae is one of the common pathogens of community‐acquired pneumonia in children.^1^ It is estimated that Mycoplasma pneumoniae pneumonia (MPP) accounts for approximately 10% to 40% of community‐acquired pneumonia in hospitalized children.^2^ The number of cases of MPP complicated by thromboembolism has increased.^3^ Refractory patients were defined as cases showing clinical and radiological deterioration despite appropriate antibiotic therapy for 7 days or more, and this is defined as refractory MPP (RMPP).^4^ If left untreated, patients with RMPP are prone to complications such as atelectasis, lung consolidation, and pleural effusion.^5^ RMPP can cause sequelae in children, the presence of which often suggests a poor prognosis.^6^ Therefore, it is important to identify and diagnose RMPP versus general MPP (GMPP) as early as possible to avoid delayed treatment of RMPP.

The incidence of RMPP has increased globally.^7^, ^8^ Excessive cytokines and highly activated cell‐mediated host immune response and resistance to macrolides by Mycoplasma pneumoniae have been reported to be associated with RMPP pathogenesis.^9^ Correlations between cytokines, chemokines or other inflammatory biomarkers and RMPP have been identified.^10^, ^11^ In addition, clinical markers such as C‐reactive protein (CRP), lactate dehydrogenase (LDH), and procalcitonin (PCT) are usually elevated.^12^, ^13^ B7‐H3, B7‐H4, and B7‐DC are all members of the immunoglobulin superfamily. B7‐H3, has a dual role in the immune system, acting as a co‐stimulatory molecule to promote immune responses, and as a co‐inhibitory molecule to suppress immune responses.^14^ As a co‐stimulatory molecule, B7‐H3 induces cellular immunity, promotes the secretion of cytokines such as IFN‐γ and IL‐8, and enhances the cytotoxicity of CD8^+^ T cells.^15^ However, it also inhibits T‐lymphocyte infiltration, thereby allowing tumors to escape immune surveillance.^16^ B7‐H4 can suppress the immune response by inhibiting the proliferation and activation of T cells through binding to receptors on T cells.^17^ Regarding B7‐DC, as a co‐stimulatory molecule, it interacts with receptors on the surface of T cells (e.g., PD‐1), and the binding of B7‐DC to PD‐1 inhibits TCR‐mediated T‐cell proliferation and cytokine production and down‐regulates T‐cell activity. It has been previously shown that serum B7‐DC is elevated in children with RMPP.^18^ Bronchoalveolar lavage (BAL) has been clinically reported to be effective in relieving inflammatory responses and improving immune function and pulmonary ventilation in children with RMPP.^19^ BAL is well‐tolerated in the treatment of RMPP with large lung foci.^20^

Timely and effective antimycoplasma therapy and immunomodulatory therapy are the main strategies for treating RMPP. To better understand the characteristics of RMPP so that early diagnosis and rational treatment can be achieved and complications due to untimely diagnosis and treatment can be reduced, a tool is needed to help predict RMPP and to guide clinical protocols for the treatment of RMPP. We hypothesized that the concentrations of soluble B7‐H3 (sB7‐H3), soluble B7‐H4 (sB7‐H4) and soluble B7‐DC (sB7‐DC) are elevated in BALF in children with RMPP, and that they may predict the risk of progression to RMPP. Therefore, we conducted this study to reveal the relationship between sB7‐H3, sB7‐H4 and sB7‐DC and RMPP.

## 2 MATERIALS AND METHODS

### 2.1 Study population

This study was a prospective cohort study. Children diagnosed with MPP in Beijing Friendship Hospital, Capital Medical University from January 2022 to May 2024, who required bronchoscopy or treatment, were selected. Treatment was performed according to the international guidelines for the management of community‐acquired pneumonia in children.^21^ Children were divided into the GMPP group and RMPP group according to the diagnostic criteria for RMPP as suggested by the Expert consensus on diagnosis and treatment of Mycoplasma pneumoniae pneumonia in children (2015).^4^

Inclusion criteria: (1) Children were over 3 months and <14 years old; (2) MPP was diagnosed by typical clinical features such as pneumonia and/or imaging changes, combined with positive results for MP pathogenicity that meet one or both of the following criteria^22^: (1) serum MP antibody potency ≥1:160 (particle agglutination test): during the course of the disease, the titer of serum MP antibody increased more than 4‐fold. (2) Positive polymerase chain reaction; and (3) In addition to the above inclusion criteria, RMPP was diagnosed when children diagnosed with MPP continue to have elevated body temperature, worsening clinical symptoms and pulmonary imaging manifestations, and extrapulmonary complications after 7 or more days of treatment with macrolide antibiotics.

Exclusion criteria: (1) children with immunodeficiency who had used immunomodulators (such as corticosteroids, cyclophosphamide, etc.); (2) children with tuberculosis infection or other mixed pathogen infections with clinical manifestations; (3) children with underlying cardiopulmonary diseases such as bronchopulmonary dysplasia and congenital heart disease; and (4) children with incomplete clinical data.

**FIGURE 1:**
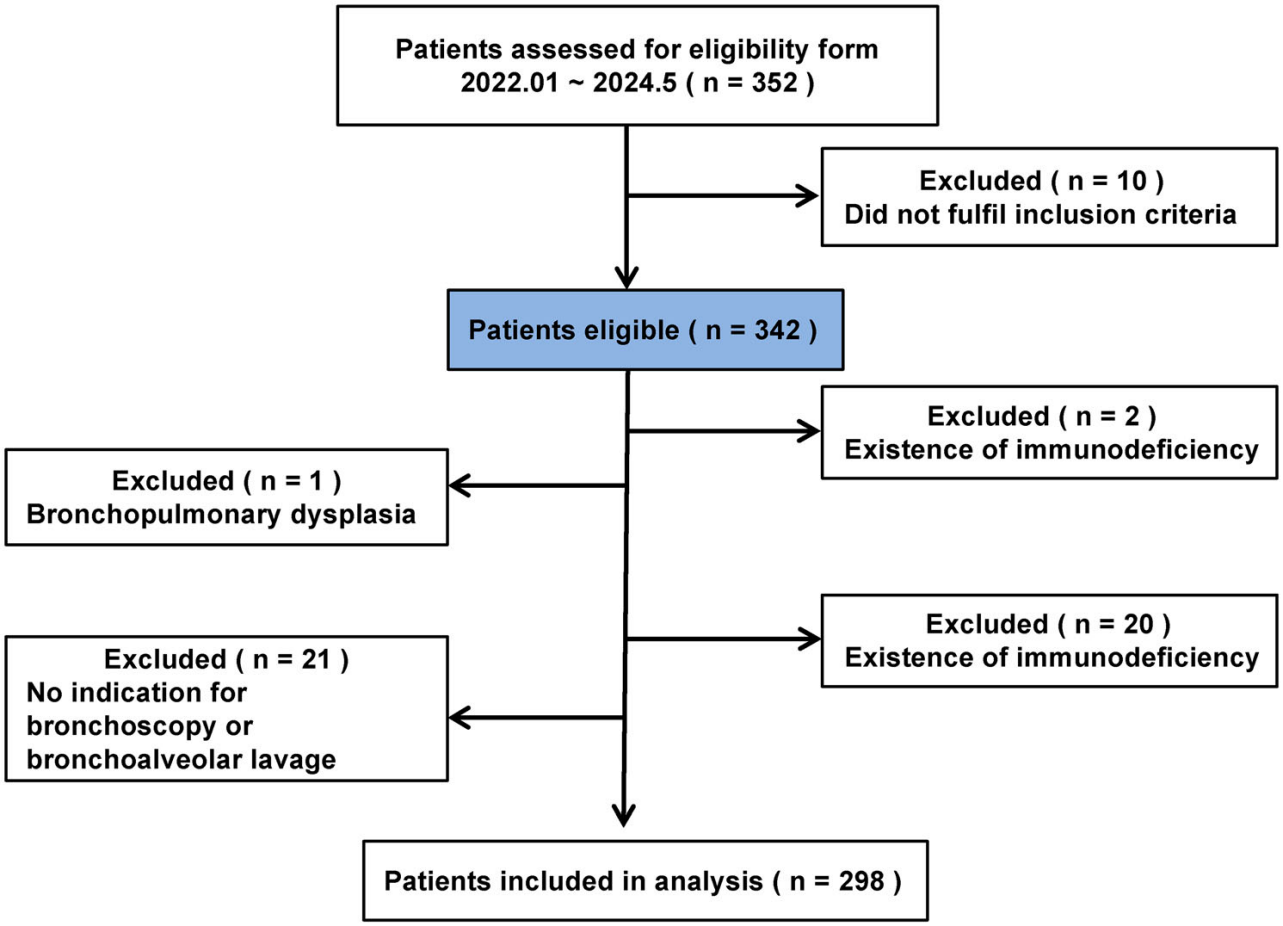
Subject enrollment process diagram.

All children were included with an informed consent signed by their guardians. Finally, 298 children were included (213 in the GMPP group and 85 in the RMPP group). The enrollment of patients is shown in Figure 1. The study was approved by the Medical Ethics Committee of Beijing Friendship Hospital, Capital Medical University (Ethics Committee No: 2021BF06‐013).

### 2.2 Clinical data

General clinical data were collected, such as age, gender, body mass index (BMI), pre‐admission course of illness, and hyperthermia (≥39.1°C) at admission. All children received chest auscultation and chest CT for the duration of their illness for findings including fine rales, lung wheezing, pleural effusion, lung consolidation, and pulmonary atelectasis. Venous blood was drawn on day 2 of admission for white blood cells (WBC), neutrophil percentage (NEP), neutrophil/lymphocyte ratio (NLR), platelets (PLT), hemoglobin (Hb), albumin, immunoglobulin (Ig)‐A/G/E, CRP, alanine aminotransferase (ALT), aspartate aminotransferase (AST), LDH, and PCT.

### 2.3 Treatment regimen

All patients were treated with anti‐MP therapy as per the standard. Azithromycin, 10 mg/(kg.day), qd, was preferred for about 7 days. After an interval of 3–4 days, a second course was started if necessary. Doxycycline, levofloxacin, and moxifloxacin were given accordingly. Other treatments included bronchoscopic intervention, glucocorticoid therapy, intravenous immunoglobulin G therapy, and anticoagulation.

### 2.4 BALF specimens

All of these children had indications for bronchoscopy (RMPP confirmation and airway lesions) and bronchoalveolar lavage (mucus plugs leading to atelectasis, plasmablastic bronchiolitis, and pathogenetic examination). Fiberoptic bronchoscopy was performed using Olympus fiberoptic bronchoscope within 2 to 5 days of the child's admission (acute phase). Bronchoscopy and BALF collection procedures were performed in accordance with the Guideline of pediatric flexible bronchoscopy in China (2018 edition).^23^ Sedation with midazolam at 0.1–0.15 mg/kg and 1–2 drops of Ephedrine Hydrochloride and Nitrofurazone Nasal Drops 5–10 min before surgery were used to make vasoconstriction, and then surface anesthesia was carried out by spraying the pharynx with Lidocaine Aerosol. Fiberoptic bronchoscopy was inserted through the nasal cavity, and each part of the airway was observed in turn. The trachea, bronchial mucosal lesions, and the location and degree of mucus plugs were observed. The mucus plugs were removed with a cell brush, and bronchopulmonary lavage was performed in combination with chest CT or chest X‐ray. The sterile saline at 37°C was used for lavage, and the amount of lavage was 0.5 ml/kg for three times. The recovery amounts were 20%, 20% and 10%, respectively. Three times of the recovered lavage fluid were mixed, of which 2/3 was used for laboratory pathogenic examination, and the remaining 1/3 was used for biomarker examination with the consent of the guardian.

### 2.5 ELISA

Human B7‐H3/CD276, human B7‐H4 ELISA and human programmed death‐2 ligand (PD‐L2, B7‐DC) ELISA kits produced by Huamei Bioengineer Company (Wuhan, China) were used. The absorbance of each sample was measured, and the optical density (OD) was measured at 450 nm using 630 nm as the calibration wavelength. The concentration of the standard substance was used as the abscissa, and the OD value was the ordinate. The software ELISACalc was used to fit the four parameters. According to the OD value of the sample, the fitting concentration was calculated from the standard curve, multiplied by the dilution factor. The total amount of lavage/actual recovery amount was calculated to obtain the concentration of the sample.

### 2.6 Data analyses

Statistical analyses were performed using SPSS 20.0 software. Data normality was checked using Shapiro–Wilk test. For normal distribution, measurement data were shown as mean ± SD and compared by Student's t‐test; for skewed distribution, data were shown as median (IOR), and Mann–Whitney *U*‐test was used for between‐group comparisons. Count data were expressed as frequency (*n*) and rate (%), and chi‐square test was used. A prediction model for RMPP was constructed, and the factors with statistical differences of *p* < 0.01 were used as independent variables to enter regression analyses. The relationship between sB7‐DC and the risk of RMPP was assessed by the restricted cubic spline (RCS) using the software package 4.0.5 in R language. Four nodes were taken according to their percentile distributions (P5, P35, P65, and P95). The factors with univariate analysis at *p* < 0.01 were taken as the independent variables, and multifactorial logistic regression was used to analyze the independent risk factors of RMPP and to construct the base model. sB7‐DC was entered in the base model to construct a compound model for predicting RMPP. The predictive value of the model was evaluated by plotting the receiver operating characteristic curve (ROC) and calculating the area under the curve (AUC). Column line graphs were plotted to assess the contribution of each independent variable to the outcome event. The model was validated using calibration curve graphs and Hosmer–Lemeshow test (HL test). A *p* value >0.05 indicated goodness‐of‐fit. Decision curve analysis (DCA), with the net benefit rate as the vertical coordinate and the high‐risk threshold as the horizontal coordinate, was plotted. The training and validation sets were generated in a ratio of 7:3 by a random function in Microsoft Excel, and the random numbers generated were ranked from smallest to largest.

## 3 RESULTS

### 3.1 Clinical characteristics and laboratory parameters of the children

A total of 298 children with MPP, including 85 with RMPP, were included. Table 1 shows the clinical characteristics of children with GMPP and RMPP. No statistical differences were observed between the two groups in terms of age, gender, BMI, and pre‐admission course of illness (all *p* > 0.05). The proportion of hyperthermia in the RMPP group was slightly higher than that in the GMPP group (49.41% vs. 38.50%, *p* = 0.084), although no statistical difference was observed. The proportion of children in the RMPP group with pleural effusion (35.29% vs. 19.25%, *p* = 0.003), lung consolidation (51.76% vs. 30.99%, *p* = 0.001), and pulmonary atelectasis (23.53% vs. 10.33%, *p* = 0.003) were significantly higher in the GMPP group. On routine blood tests, no statistical differences were observed for any of the indicators, including WBC, NEP, NLR, PLT, Hb, and albumin (all *p* > 0.05). Compared with the GMPP group, the RMPP group had a statistically significant difference in CRP (52.36 [29.01, 82.98] vs. 45.60 [31.64, 57.16], *p* < 0.001) and AST (32.36 [14.30, 46.36] vs. 22.99 [14.17, 30.80], *p* < 0.001) (Table 2).

**TABLE 1:** Clinical characteristics of children with GMPP and RMPP.

Variables	GMPP (*n* = 213)	RMPP (*n* = 85)	*p* value
*General characteristics*			
Age, years	6.2 ± 2.7	6.0 ± 2.2	0.865
*Gender*, *n* (*%*)			0.986
Female	105 (49.30)	42 (49.41)	
Male	108 (50.70)	43 (50.59)	
Body mass index (kg/m^2^)	15.6 [14.0, 17.9]	15.4 [14.3, 17.6]	0.756
Pre‐admission course of illness (days)	7.0 ± 1.9	7.4 ± 2.5	0.125
*Signs and symptoms*			
High‐grade fever, *n* (%)	82 (38.50)	42 (49.41)	*0.084*
Fine rales, *n* (%)	65 (30.52)	28 (32.94)	0.683
Lung wheezing, *n* (%)	52 (24.41)	28 (32.94)	0.134
Pleural effusion, *n* (%)	41 (19.25)	30 (35.29)	*0.003*
Lung consolidation, *n* (%)	66 (30.99)	44 (51.76)	*0.001*
Pulmonary atelectasis, *n* (%)	22 (10.33)	20 (23.53)	*0.003*

*Note*: All data are shown as median [IQR] or *N* (%). Categorical values were compared using the chi‐square test. Student's *t*‐test or Mann–Whitney test was used to assess differences between the two groups. *p* < 0.05 was considered statistically significant.

**TABLE 2:** Routine laboratory indicators in children with GMPP and RMPP.

Variables	GMPP (*n* = 213)	RMPP (*n* = 85)	*p* value
*Routine blood*			
WBC (×10^9^/L)	6.6 [5.2, 11.5]	6.3 [5.3, 10.5]	0.452
Neutrophil percentage (%)	4.2 [2.6, 6.7]	4.0 [2.7, 6.5]	0.924
NLR	1.77 [1.25, 3.12]	1.92 [1.21, 2.95]	0.972
PLT (×10^9^/L)	273.3 ± 54.6	288.5 ± 63.96	0.153
Hb (g/L)	120 ± 15	114 ± 16	0.108
Albumin (g/L)	41.5 ± 4.6	38.5 ± 4.9	0.192
*Humoral immunity*			
Total IgG (g/L)	7.24 ± 3.69	8.00 ± 4.10	0.125
Total IgA (g/L)	0.90 ± 0.64	1.08 ± 0.89	0.252
Total IgE (g/L)	95.5 [45.8, 164.3]	61.5 [30.3, 175.3]	0.21
*Biochemical indicators*			
CRP (mg/L)	45.60 [31.64, 57.16]	52.36 [29.01, 82.98]	<*0.001*
ALT (U/L)	32.36 [23.36, 42.36]	39.94 [20.35, 45.05]	0.53
AST (U/L)	22.99 [14.17, 30.80]	32.36 [14.30, 46.36]	<*0.001*
LDH (U/L)	359.75 ± 234.62	403.02 ± 214	0.761
PCT (ng/ml)	0.29 [0.22, 0.38]	0.30 [0.25, 0.43]	0.724

*Note*: All data are shown as median [IQR]. Student's *t*‐test or Mann–Whitney test was used to assess differences between the two groups. *p* < 0.05 was considered statistically significant.

Abbreviations: ALT, alanine aminotransferase; AST, aspartate aminotransferase; CRP, C‐reactive protein; Hb, Hemoglobin; Ig, immunoglobulin; LDH, lactate dehydrogenase; NEP%, neutrophil percentage; NLR, neutrophil/lymphocyte ratio; PCT, procalcitonin; PLT, platelets; WBC, white blood cells.

### 3.2 sB7‐H3, sB7‐H4 and sB7‐DC in BALF

Table 3 observes higher levels of sB7‐H3 and sB7‐H4 than sB7‐DC in children with MPP. No statistically significant difference was found between sB7‐H3 and sB7‐H4 in the RMPP group and GMPP group (both *p* > 0.05). Notably in the RMPP group, the levels of sB7‐DC were significantly higher than in the GMPP group (91.66 [77.36, 122.5] vs. 64.87 [47.07, 86.46], *p* < 0.001).

**TABLE 3:** sB7‐H3, sB7‐H4 and sB7‐DC in BALF of children with GMPP and RMPP.

	GMPP (*n* = 213)	RMPP (*n* = 85)	*p*
sB7‐H3 (pg/ml)	229.87 [179.71, 271.80]	238.72 [166.06, 296.84]	0.339
sB7‐H4 (pg/ml)	241.50 [189.20, 221.30]	253.01 [221.30, 272.1]	0.129
sB7‐DC (pg/ml)	64.87 [47.07, 86.46]	91.66 [77.36, 122.5]	<*0.001*

*Note*: All data are shown as median [IQR]. Mann–Whitney test was used to assess the difference between the two groups. *p* < 0.05 was considered statistically significant.

### 3.3 Dose–response analysis of sB7‐DC and RMPP

**FIGURE 2:**
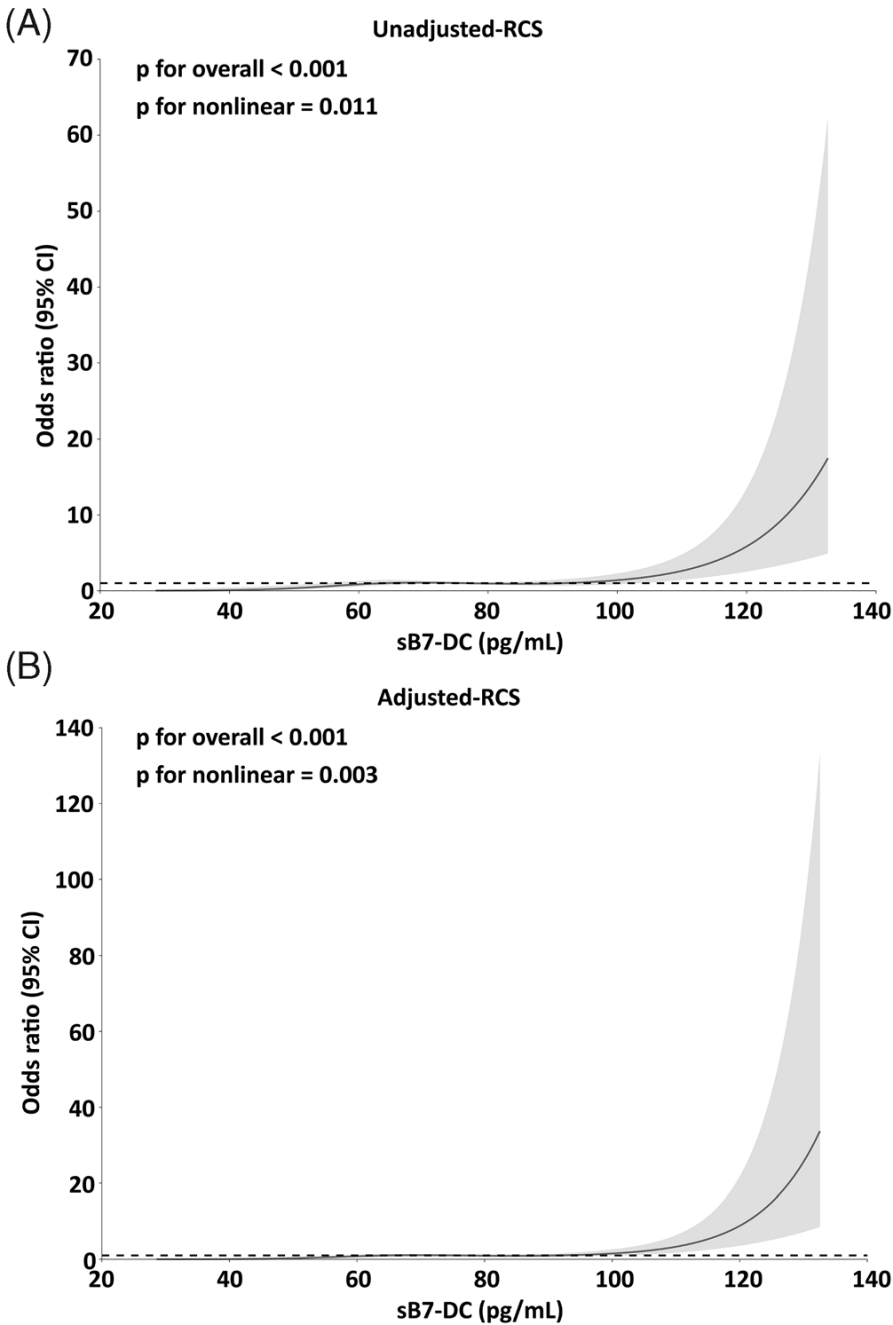
Analysis of the dose–response relationship between sB7‐DC and RMPP occurrence based on a RCS model (4 nodes). (A) Unadjusted RCS plot. (B) RCS plot adjusted for age and gender as confounders. *p*_for overall_ usually refers to the *p* value of the overall effect of the model, which is used to test whether the joint effect of all independent variables in the model (including both linear and nonlinear terms) on the dependent variable is significant. *p*_for nonlinear_, on the other hand, refers specifically to the *p* value of the nonlinear part of the model, which is used to test the significance of the nonlinear relationship between the independent and dependent variables. *p* < 0.05 was considered statistically significant.

As shown in Figure 2, we investigated the dose (sB7‐DC)‐response (RMPP) relationship using RCS analysis with a combination of spline functions and logistic regression, with the number of nodes for curve fitting set to four (29.28, 60.37, 85.77, 126.9). Univariate logistic regression of the RCS showed a nonlinear dose–response relationship between continuous changes in sB7‐DC and RMPP (*p*_for nonlinear_ = 0.011), and that sB7‐DC was roughly positively correlated with the occurrence of RMPP, especially when sB7‐DC was >76.505 pg/ml. After adjusting for age and gender, there was still a non‐linear dose–response relationship between the continuous changes of sB7‐DC and RMPP (*p* = 0.003).

### 3.4 Risk factor analysis for RMPP

Next, we analyzed the independent factors associated with the risk of developing RMPP by logistic regression, as shown in Table 4. Six variables were included in the logistic regression analysis by obtaining variables with *p* < 0.01 in the univariate analysis, including high‐grade fever, pleural effusion, lung consolidation, pulmonary atelectasis, CRP, and AST. Age and gender were taken as confounders to construct the base model for predicting RMPP. Finally, pleural effusion, lung consolidation, pulmonary atelectasis, and CRP were identified as independent risk factors for RMPP (Figure 2A). Furthermore, we incorporated sB7‐DC into the above base model to construct a compound model. The entry of sB7‐DC significantly made AST become significant for predicting the risk of RMPP. However, the efficacy of lung consolidation and pulmonary atelectasis as independently associated risk factors for RMPP was canceled (Figure 2B).

**TABLE 4:** Independent risk factors associated with RMPP by multifactorial logistic analysis.

Base model					Compound model			
Variables	β	SE	*p*	OR (95%CI)	β	SE	*p*	OR (95%CI)
Intercept	−3.63	0.58	<*0.001*	0.03 (0.01 ~ 0.08)	−8.75	1.31	<*0.001*	0.00 (0.00 ~ 0.00)
*Pleural effusion*								
0				1.00 (Reference)				1.00 (Reference)
1	1.11	0.38	*0.004*	3.04 (1.43 ~ 6.45)	1.17	0.45	*0.01*	3.22 (1.33 ~ 7.81)
*Lung consolidation*								
0				1.00 (Reference)				1.00 (Reference)
1	1.19	0.35	<*0.001*	3.28 (1.64 ~ 6.55)	0.65	0.42	0.12	1.91 (0.85 ~ 4.33)
*Pulmonary atelectasis*								
0				1.00 (Reference)				1.00 (Reference)
1	0.91	0.43	*0.035*	2.50 (1.06 ~ 5.85)	0.56	0.52	0.286	1.75 (0.63 ~ 4.87)
CRP	0.03	0.01	*<0.001*	1.03 (1.01 ~ 1.04)	0.03	0.01	*0.007*	1.03 (1.01 ~ 1.05)
AST	0.02	0.01	0.067	1.02 (1.00 ~ 1.04)	0.05	0.02	*0.002*	1.05 (1.02 ~ 1.08)
sB7‐DC					0.05	0.01	*<0.001*	1.05 (1.04 ~ 1.07)

*Note*: Base model, incorporating clinical characteristics and routine laboratory indicators for analysis. sB7‐DC on the base model was entered into the composite model. *p* < 0.05 was considered statistically significant.

### 3.5 Column line graph and efficacy of predictive models

**FIGURE 3:**
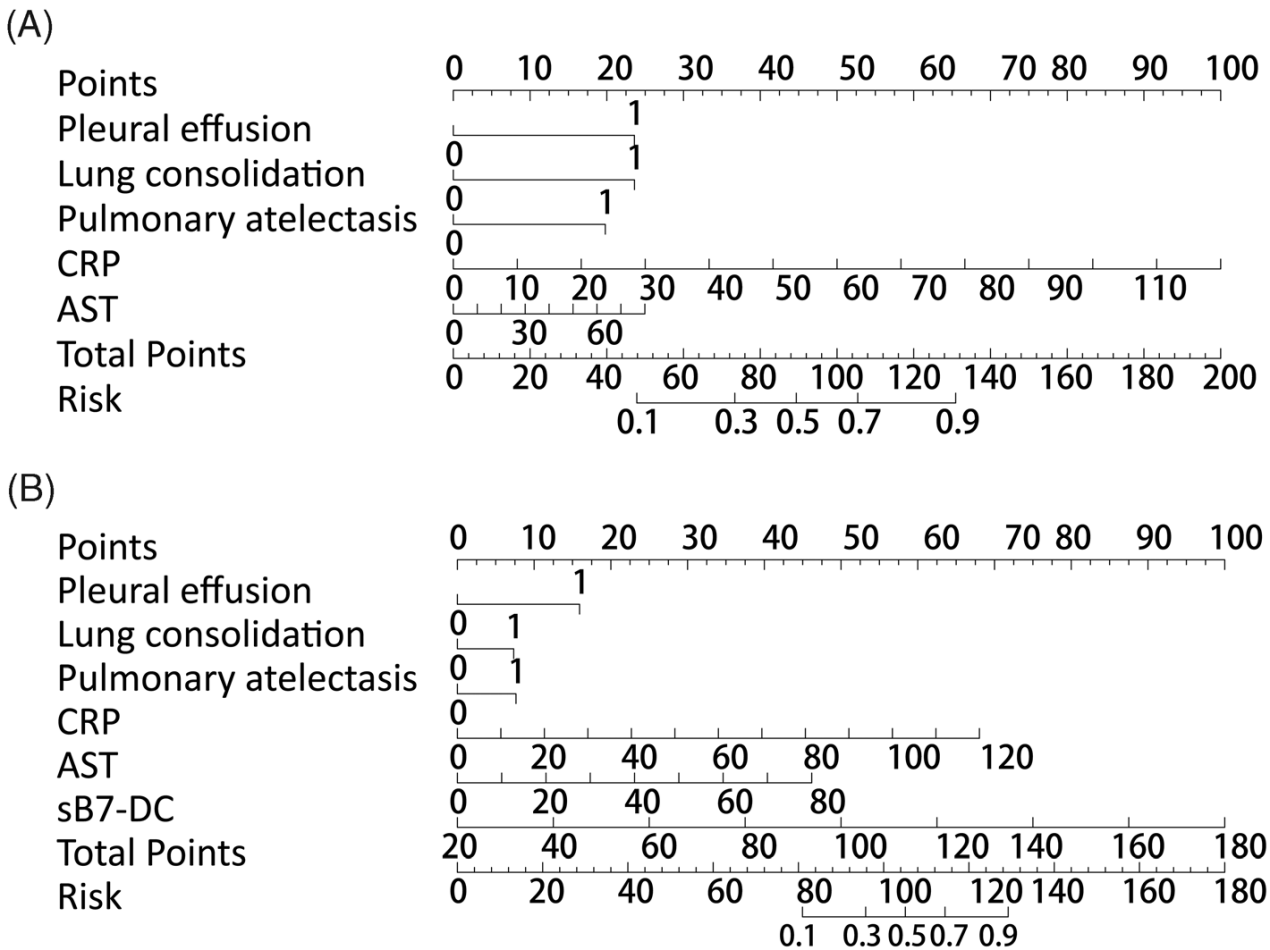
Column line plots of predicted child RMPP. (A) Column line graph for the base model. (B) Line plots for the compound model. The line‐column plot consists mainly of the variable names on the left and the scaled line segments on the right, the length of which reflects the magnitude of the factor's contribution to the outcome event.

Based on the multifactorial logistic regression results described above, the contribution of independent correlates to the prediction of RMPP was visualized using a column‐line diagram, as shown in Figure 3. In the base model, the total scores of pleural effusion, lung consolidation, pulmonary atelectasis, and CRP were associated with an increased risk of RMPP. When pleural effusion, lung consolidation and pulmonary atelectasis were present and CRP was 70 mg/L in BALF, the scores were 22, 22, 20, and 50, respectively, with a total score of 102, corresponding to a risk value for RMPP >0.7 (Figure 3A). In the compound model, the predictive efficacy of lung consolidation and pulmonary atelectasis was canceled, and the contribution of sB7‐DC to the prediction of RMPP was of higher value (Figure 3B).

**FIGURE 4:**
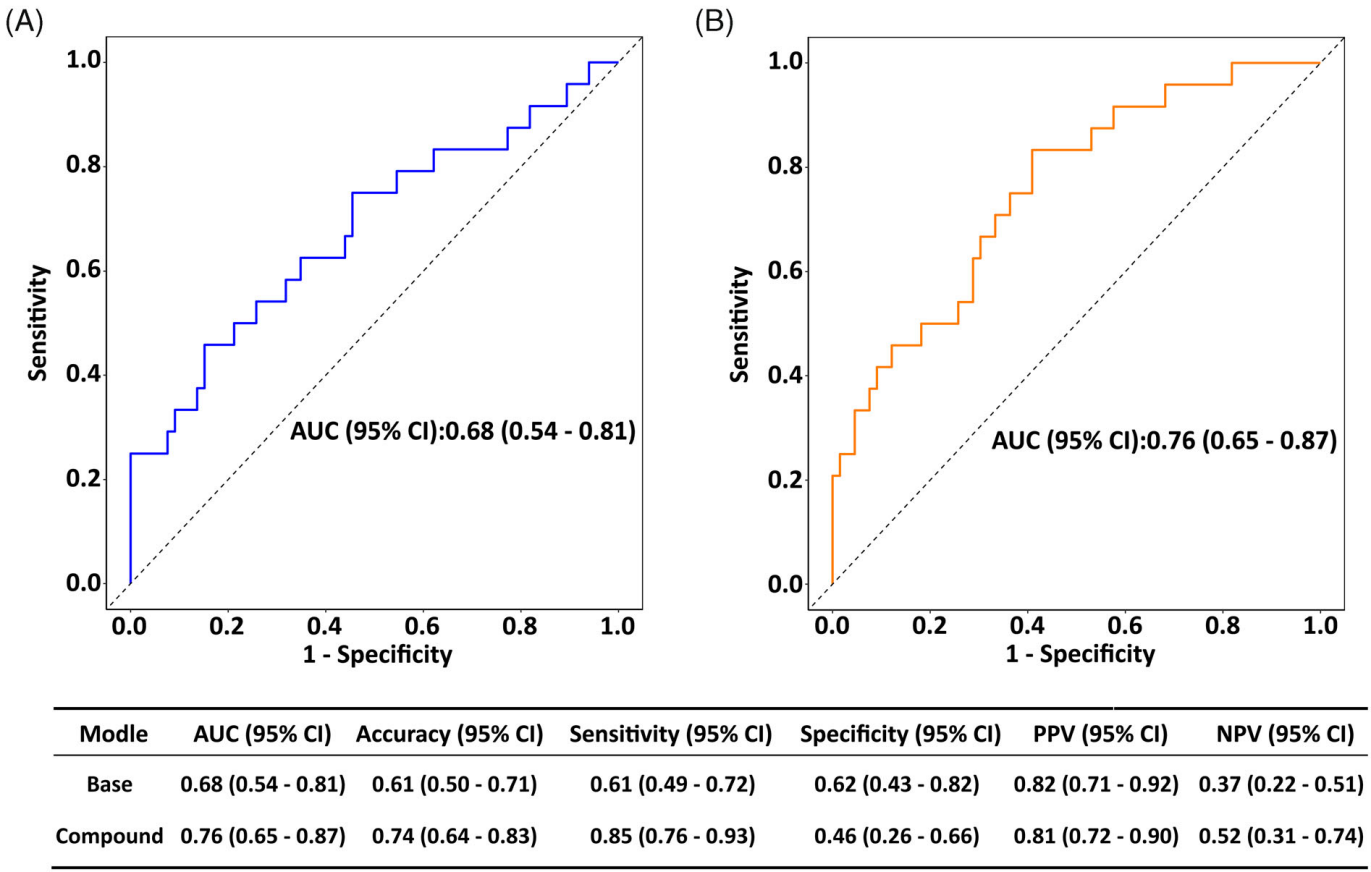
ROC curves of the base model and compound model for predicting RMPP in children. (A) ROC curve for the base model. (B) ROC curve for the compound model. AUC, area under the curve; NPV, negative predictive volume; PPV, positive predictive volume.

**FIGURE 5:**
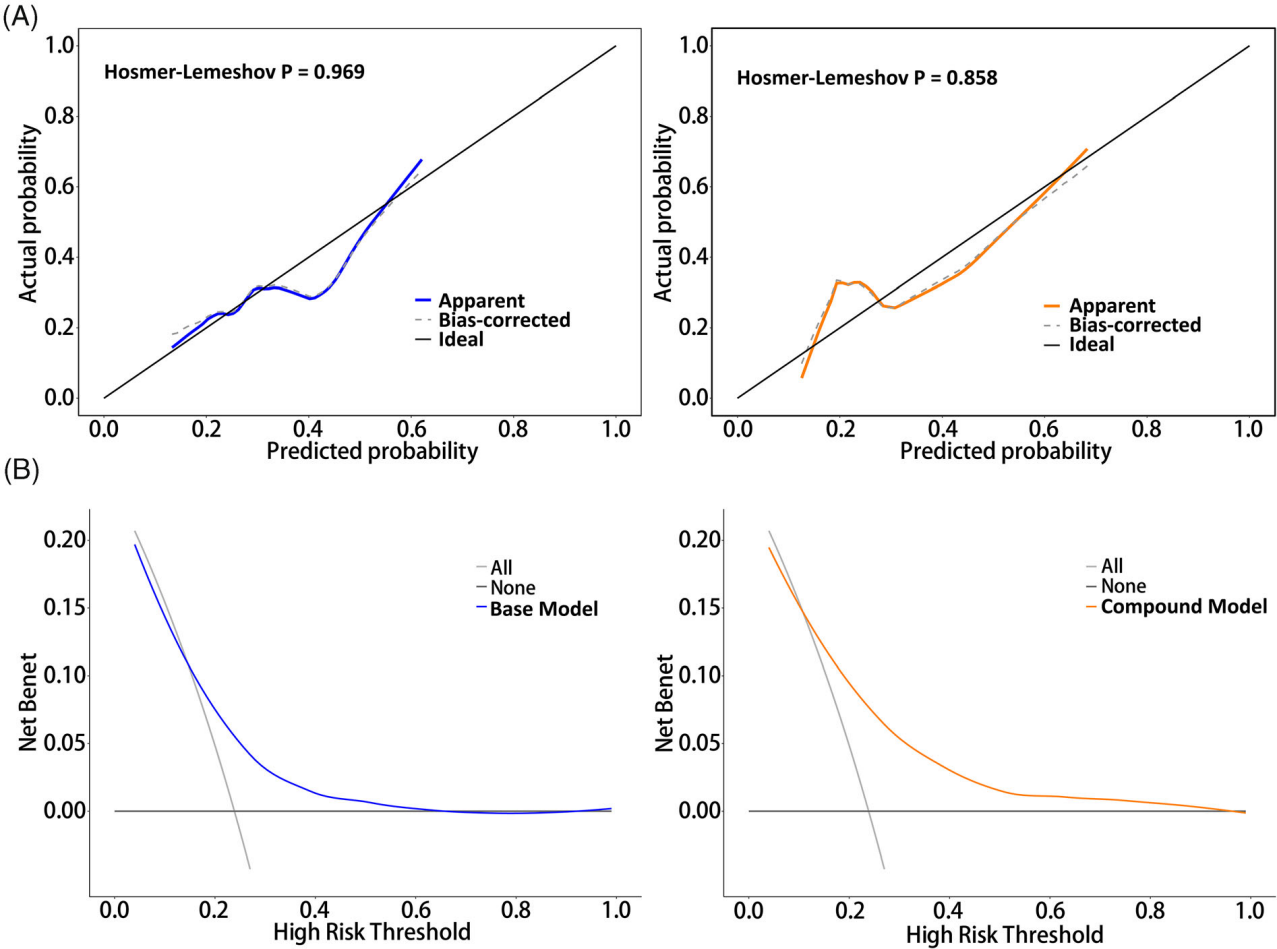
Model efficacy analysis. (A) Calibration curves of the base model versus the compound model. (B) DCA of the base model versus the compound model.

The predictive models were plotted by ROC curves (Figure 4, all *p* < 0.05). Based on the AUC of the two models, the compound model had higher predictive efficacy than the base model (*p* = 0.045). Notably, the specificity and sensitivity of the compound model were higher than those of the base model. In addition, the accuracy of model prediction was assessed by plotting a calibration curve (Figure 5A). Both the base model and the compound model presented a good model fit according to the HL test (all *p* > 0.05). Finally, the net benefit of RMPP risk was observed using DCA plots (Figure 5B). In the base model, net benefits were >0 (for 0.1) when the high risk threshold was close to 0.2; and the smaller the value taken between 0.2 and 0.6, the greater the net benefits. The compound model showed that net benefits were close to 0.15 for a high risk threshold of 0.1. Thus, the net benefits increased for the compound model compared to the base model.

## 4 DISCUSSION

Children with RMPP often have a poor prognosis and a significantly prolonged course of disease after macrolide therapy.^24^ These children are at risk of multiple complications in the acute phase, while their lung structure or function may be severely compromised, leading to long‐term complications.^9^ Immune dysfunction may mediate the progression of MPP to RMPP.^25^ The main results of this study were that there was a nonlinear dose–response relationship between sB7‐DC and the occurrence of RMPP; the RMPP prediction model constructed on the basis of clinical features and biochemical indicators had good diagnostic performance and net benefits (high risk threshold between 0.2 and 0.6), and the entry of sB7‐DC into the base model increased the accuracy and sensitivity of the base model and had higher net benefits (high risk threshold between 0.1 and 0.9).

In this study, the proportion of children with hyperthermia or adverse pulmonary imaging features (pleural effusion, lung consolidation, and pulmonary atelectasis) and biochemical indices (CRP and AST) levels were significantly higher in the GMPP group. Elevated CRP, LDH, AST, and PCT are predictors of RMPP.^26^ Our study did not observe significant differences in LDH and PCT levels between the RMPP and GMPP groups. This may be related to patient regional selection or stronger regional integration. Shen et al. developed a predictive model for RMPP using CRP, LDH, and D‐dimer and visualized the risk of RMPP using a column‐line graph to identify RMPP in the early stage.^27^ Our study also constructed a predictive model (base model) for RMPP using significantly different clinical features and biochemical indicators. The results showed that pleural effusion, lung consolidation, pulmonary atelectasis, and CRP were all independent risk factors for predicting RMPP, with CRP contributing the most to the model. CRP was a commonly used acute‐phase non‐specific inflammation marker. It eliminates pathogenic microorganisms and damaged, necrotic and apoptotic cells by activating complement and enhancing phagocytosis. CRP is characterized by early sensitivity to infection‐induced inflammation or tissue damage and rapid recovery as the lesion subsides and the tissue structure and function return.^28^ However, despite the predictive efficacy of the model, the low AUC of 0.68 (0.54–0.81) may not be applicable in some cases, leading to clinical practitioners ignoring the severity of the disease or over‐treatment. Therefore, there is a need to develop a column chart that can predict RMPP more accurately.

Most children with RMPP experience increased airway secretions and mucus plugs, leading to a range of disorders such as poor airway ventilation, obstruction, and even pulmonary atelectasis. For the pharmacological treatment of RMPP, azithromycin is the drug of choice for treating pneumonia caused by mycoplasma infection.^29^ However, for RMPP patients, azithromycin may not be able to fully control the condition, leading to its persistence or worsening. In such cases, doctors need to consider other treatment options, one of which is doxycycline.^30^ Doxycycline should only be used after 72 h of azithromycin treatment, and it's important to note that doxycycline is not without risks. It may cause yellowing of teeth and poor enamel development, so it is only suitable for children over 8 years old.^31^ However, a survey among Native American children suspected of having Rocky Mountain spotted fever revealed that the use of doxycycline did not lead to noticeable tooth staining.^30^ We speculate that the effectiveness of doxycycline may be influenced by factors such as race, drug dosage, and treatment duration. Additionally, doxycycline may also induce gastrointestinal adverse reactions, including nausea, vomiting, abdominal pain, and diarrhea.^32^ The use of BLA technique can effectively alleviate the above problems. The present study assessed the diagnostic value of RMPP by measuring the concentration of factors in BALF. Our study showed a significant increase in the concentration of sB7‐DC in BALF in children with RMPP. B7‐DC has been reported to share the programmed cell death protein 1 receptor with B7‐H1.^32^ In disease models involving adjuvant Th2‐dependent cytokine production and B7‐DC, anti‐B7‐DC monoclonal antibodies exacerbate the disease, suggesting that B7‐DC is the primary mediating ligand inhibitor.^33^, ^34^ B7‐DC promotes targeted migration and activation of immunosuppressive to the lungs.^33^ An imbalance in the Th1/Th2 immune response in MPP may result in the inability to effectively clear pathogens while triggering excessive inflammatory responses and tissue damage.^35^ Furthermore, Th2 mediates the expression of B7‐DC through the production of IL‐4 and IL‐13.^36^ Therefore, B7‐DC may mediate the development of GMPP by participating in the Th2 cytokine response. Based on the fact that sB7‐DC concentration was significantly elevated in children with RMPP, we first examined the dose–response relationship between sB7‐DC and RMPP using RCS analysis combining spline function and logistic regression. Subsequently, sB7‐DC was entered into the base model to construct a compound model. Subsequently, based on the AUC of the ROC curve and the net benefit of the DCA plot, it was observed that sB7‐DC improved the predictive efficacy of the base model to 0.76 (0.65–0.87), with a wide range of values of high risk threshold for a net benefit >0, which suggests that (1) the model is able to provide valuable predictive information; (2) it is clinically useful, aiding in informed medical decision‐making; and (3) the model is insensitive to small changes, and is able to maintain a relatively stable predictive performance in different situations.

This study has some limitations. BALF was not easily available due to ethical requirements. Therefore, specimens from children without MPP were not obtained for this study. Although we have internally validated the column line graph within the same medical center, more prospective multi‐center studies are needed. Additionally, some children were administered medications before BLA, which might have affected the measurements.

## 5 CONCLUSION

In conclusion, serum CRP and sB7‐DC in BLAF are independent risk factors for RMPP. The column‐line diagram can effectively and quantitatively predict the risk of RMPP, and more accurately and conveniently guide clinicians to identify RMPP and make medical decisions.

## CONFLICT OF INTEREST STATEMENT

The authors have no conflicts of interest to declare.

## ETHICS STATEMENT

The present study was approved by the Ethics Committee of Beijing Friendship Hospital, Capital Medical University (Ethics Committee No: 2021BF06‐013) and written informed consent was provided by all patients prior to the study start. All procedures were performed in accordance with the ethical standards of the Institutional Review Board and The Declaration of Helsinki, and its later amendments or comparable ethical standards.

## Data Availability

The data that support the findings of this study are available from the corresponding author upon reasonable request.
